# Piglets' acute responses to local anesthetic injection and surgical castration: Effects of the injection method and interval between injection and castration

**DOI:** 10.3389/fvets.2022.1009858

**Published:** 2022-09-29

**Authors:** Mathilde Coutant, Jens Malmkvist, Marianne Kaiser, Leslie Foldager, Mette S. Herskin

**Affiliations:** ^1^Department of Animal Science, Aarhus University, Tjele, Denmark; ^2^Bioinformatics Research Centre, Aarhus University, Aarhus, Denmark

**Keywords:** local anesthesia, castration, pig, pain, stress, acute responses

## Abstract

Although applied in some countries, efficacy of local anesthetics based on procaine to mitigate acute responses to piglet castration remains questioned. This paper presents results from a factorial study examining the effects of two methods of injection of a procaine-based drug (intra-funicular, IF, vs. intra-testicular, IT), and four intervals between drug injection and castration (2.5, 5, 10, and 30 min) on acute responses of 3–4 day old piglets. The study involved 597 male piglets, and 13 treatments: surgical castration without anesthesia (CC), local anesthesia followed by castration involving all combinations of injection method and interval, and sham handling separated by the same four intervals (SH). Responses of piglets to drug injection, castration and sham handling were evaluated based on quantification of intra-procedural vocalizations and leg movements, as well as saliva cortisol concentration in samples taken before and after castration. No differences were found between IF and the simpler IT injection method. Intervals of 2.5 or 30 min led to stronger piglet responses than the other intervals. Overall, treatments involving anesthesia led to significantly stronger responses than sham handling, during both injection and castration. All treatments, even sham handling, led to a significant increase in saliva cortisol, with no differences between anesthesia treatments and controls. Based on these results, castration 5–10 min after intra-testicular injection of procaine seems to be preferable as compared to the other treatments tested. However, piglets still showed measurable signs of pain and stress during both injection and castration, while handling alone (including the use of a castration bench) triggered a noticeable stress response. In light of these findings, the overall benefit of the procedure in terms of piglet welfare remains arguable.

## Introduction

Surgical castration is a routine practice that involves millions of pigs yearly in the EU and other global regions. The procedure, consisting of the removal of the male piglets' testes *via* severing of the spermatic cords, is primarily performed to prevent boar taint, perceived by humans as an unpleasant odor, and potentially present in meat from entire males (1). Implemented without pain mitigation, surgical castration leads to substantial pain and stress in piglets, as measured for example by a higher prevalence of high frequency vocalizations (2, 3), increased plasma cortisol concentrations (4–7), and in-pen behavioral alterations (5, 8, 9) compared to sham handled piglets. As a result, pain mitigating strategies such as administration of non-steroidal anti-inflammatory drugs (NSAIDs) and/or local anesthetics prior to the procedure have been developed and implemented. Studies suggest that local anesthesia limits the expression of high pitch vocalizations (2, 10, 11), reduces levels of leg movements interpreted as resistance (11–14), and decreases the plasma cortisol response (10, 15). In accordance with the ‘European Declaration on alternatives to surgical castration in pigs', stating that from 2012, all piglets should receive prolonged pain relief (16), various European countries have enforced the use of local anesthetics prior to piglet castration, and the practice is common, but not systematic, in the European pig industry (17).

In Denmark, the fourth largest producer of pigs in Europe (18), systemic pain relief using NSAIDs has been mandatory since 2009, and the administration of local anesthetics became a code-of-practice in 2019, following an initiative from the Danish pig industry (19). Farmers and herdsmen are allowed to administer the anesthetics themselves after completing a training led by a veterinarian, following the guidelines provided by the Danish Veterinary and Food Administration [DVFA, (19)]. These include administration of the anesthetic using an intra-funicular injection (injection into the spermatic cord, testis, and scrotal skin), also referred to as a “three-step” method, and a 5 to 10 min time interval between injection and castration (19). The procedure is performed using one of the two anesthetics drugs (both procaine-based) legalized for piglet castration in Denmark.

Yet, most studies investigating the efficacy of local anesthesia to mitigate piglet pain have been carried out in laboratory-like conditions, involving procedures performed by skilled veterinarians [e.g., (10, 12)]. Thereby, the procedures likely considered subtle aspects of the injection techniques such as the speed or the pressure applied on the testes, which potentially may affect the pain triggered as well as administration efficacy (20, 21). Other studies have been performed on-farm, but still involved a trained veterinarian for the administration of local anesthetic and castration (22, 23). Thus, in the absence of a comparative study, it is not known whether herdsmen, despite their mandatory training, perform complex procedures such as intra-funicular injections to a similar level as experienced veterinarians. Consequently, the possibility for diverging efficacy of the procedure when performed in practice, as compared to studies led by veterinarians, means that predictions for the actual welfare impact of castration involving local anesthesia, as commonly performed commercially, may be uncertain. A field trial setup was therefore implemented in the present study.

While the efficacy of local anesthesia administered prior to piglet castration has been mostly reported in studies using lidocaine, procaine is the active ingredient most frequently used as anesthetic for pig castration in the EU (17). Textbooks reviewing lidocaine and procaine report differences in terms of onset of action, efficacy and potency (24–26). In addition, recent studies suggested unsatisfactory pain mitigation of procaine administered alone before surgical castration of 3–7 day old piglets (12, 22, 27).

Irrespectively of the drug used, knowledge on the welfare impact of castration with prior administration of local anesthetics remains limited, as only few studies have reported piglets' response to the injection itself. As reviewed by Kongsted et al. (28), studies reporting effect of different methods of injection are also scarce. In addition, the effect of specific modalities of the anesthetic administration remain poorly documented. For instance, while the method of injection is often described with regards to the anatomical location (intra-funicular, intra-testicular), descriptions of the orientation of the needle, pressure applied on the testes, or distribution of the injected liquid is often lacking (29). Potential effects of the interval between injection of the anesthetic and castration have been discussed with regards to the onset of action, but, to our knowledge, only one study compared the impact of interval on the acute response to castration for a specific drug (30).

The aim of the present study was to compare the effect of two methods of injection of procaine as a local anesthetic and four time intervals between injection and castration on piglets' responses to injection and castration in field trial conditions resembling commercial practice. Acute responses were evaluated based on vocalizations and number of leg movements, interpreted as resistance, during injection and castration, as well as saliva cortisol concentrations before and after surgical castration.

## Materials and methods

### Animals

The experiment was carried out between July and October 2020 and was conducted in a Danish conventional sow herd with approximately 1,300 sows giving birth to (Landrace × Yorkshire) × Duroc crossbred piglets. Sows were loose-housed in farrowing pens measuring 3.1 × 2.8 m, set as a crate in the first week post-farrowing, i.e., during the experiment.

Trials were conducted 2 days per week, corresponding to the routine days of castration at the farm. Earlier in the week, litters reaching 3 to 4 days of age on the weekday of experimentation (with day 0 defined as the day of birth of the last piglet in a litter), and counting at least six males, were clinically assessed, and male piglets selected. All selected piglets were clinically healthy and free of overt anatomical malformations. Piglets weighting < 0.9 or >2.3 kg on the day of selection were not included in the study, due to a risk of improper fit in the castration bench during testing. Each of six male piglets selected within a litter was randomly assigned to one of 13 treatment groups (Table 1) according to a randomization plan balancing treatments between litters, experimental days, and experimental weeks. On the day of castration, the health status of experimental piglets and sows were re-assessed, and piglets were excluded from the experiment if the inclusion criteria were not fulfilled (severe diarrhea, lameness, or sow rectal temperature higher than 39°C).

**Table 1:** Description of the 13 treatment groups involved in the study.

	**IF02**	**IF05**	**IF10**	**IF30**	**IT02**	**IT05**	**IT10**	**IT30**	**CC**	**SH02**	**SH05**	**SH10**	**SH30**
**IM**	IF	IF	IF	IF	IT	IT	IT	IT	–	SH	SH	SH	SH
**TI**	2.5	5	10	30	2.5	5	10	30	–	2.5	5	10	30
**N**	50	50	50	49	50	49	50	49	50	50	25	25	50

Apart from CC (control castrated), all treatments were a combination of a method of injection (IM): IF, intra-funicular injection; IT, intra-testicular injection; SH, sham handling; and time interval between injection and castration (TI, min): 02, 2.5 min; 05, 5 min; 10, 10 min; 30, 30 min. N, number of piglets tested per treatment.

All piglets were identified by a number written on their back using a food-safe marker. Experimental piglets were allowed to be cross-fostered in the first days of life, but could not be moved from their litter after selection. Cross-fostering of non-experimental littermates was permitted up to the morning prior to castration. The piglets were administered a suspension of 45 mg toltrazuril and 200 mg gleptoferron (Forceris™, 1.5 mL, Ceva Animal Health A/S, Libourne, France) on day 1 after farrowing. The experimental piglets were not ear tagged, tail docked, or teeth clipped before castration. In order to avoid confounding of the results on the efficacy of the local anesthetic, piglets were administered an NSAID (intramuscular injection of 1.5 mg meloxicam; Melovem, 0.3 mL, Dopharma, The Netherlands) as analgesic after completion of the data collection and within 24 h after castration.

### Study design

On the day of castration, experimental piglets were weighed. Saliva samples were taken approximately 35 to 40 min before bringing the piglets to the testing area, a calm room outside the farrowing room. All experimental piglets plus one extra littermate selected at random were transported together in a plastic box (size: 71.5 × 53.0 × 39.5 cm) layered with straw, and placed underneath a heat lamp (averaging 20°C, ranging from 15 to 25°C at recipients) upon arrival in the testing area. Piglets were injected, castrated or sham handled one by one, respecting a randomized testing order, and following a predefined schedule ensuring that the experimental intervals between procedures were respected. During all procedures, piglets were fixated while lying on their back, in a commercially available castration bench (Unitron A/S, Kolding, Denmark). For the experimental purpose, the bench was modified to enable larger amplitudes of front leg movements, and more natural opening of the mouth during vocalizing. To further ensure a proper fit in the bench, considering the variation in piglets' body size, a soft material (5-mm yoga matt; Figure in Supplementary Figure S1) could be placed in the bench.

In-between procedures, piglets were returned to the heated box with their littermates. Immediately after castration or last sham handling, piglets were individually subjected to complementary testing not reported in the present paper, and brought back to the sow in the farrowing pen. On average 17 min after castration or last sham handling, a second saliva sample was taken in the farrowing unit. Later in the afternoon, approximately 6 h after castration, a last saliva sample was taken for cortisol determination.

In accordance with the clinical trial permit (described below), piglets were closely monitored for drug-related side effects up to 72 h after anesthetic injection and castration.

Two experimenters were present in the testing area: an experimenter performing the procedures, and an experimenter starting and stopping the recordings. These two were not blinded to the experimental treatments. All other experimenters, selecting the piglets, sampling them, recording the data and creating the datasets were blinded until the start of the statistical analysis.

### Treatments

A total of 597 piglets were assigned to one of thirteen treatments (Table 1): Surgical castration without local anesthesia involving a single stay in the bench (control-castrated; CC), intra-funicular (IF) injection of 0.5 mL of local anesthetic per testis and subsequent castration after 2.5 min (IF02), 5 min (IF05), 10 min (IF10) or 30 min (IF30), intra-testicular (IT) injection of 0.5 mL of local anesthetic per testicle and subsequent castration after 2.5 min (IT02), 5 min (IT05), 10 min (IT10) or 30 min (IT30), sham handling (SH) with two stays in the bench without tissue damage inflicted (sham anesthesia and sham castration), separated by 2.5 min (SH02), 5 min (SH05), 10 min (SH10) or 30 min (SH30).

### Procedures

All surgical and injection procedures were performed by the same experimenter, an experienced farm staff from Aarhus University trained in accordance with standards from the DVFA (19). To achieve a uniform injection technique among the experimental piglets, the experimenter received additional training led by a veterinarian and practiced the different procedures on approximately 50 piglets before experimentation. For all piglets, the precise duration of each procedure (injection, castration, sham handling) was recorded to the nearest second.

#### Anesthetic drug

The anesthetic used in the study was a procaine hydrochloride 2% solution (Procamidor^®^ Vet., 20 mg/mL, Richter Pharma AG, Wels, Austria). The product was administered using an automatic syringe (Prima Tech^®^ 0.5 mL in 0.1 mL increments) with a 25G needle for the intra-funicular injection (0.5 × 16 mm, BD Microlance™ 3, BD, New Jersey, USA). For the intra-testicular injection, in order to get the shortest needle length possible, a 26G needle (0.45 × 12 mm, Sterican^®^ Insulin needle, B Braun Medical SA, Barcelona, Spain) was used, together with a 5 mm plastic stopper. Needles were changed between each piglet.

#### Injection types

**Figure 1:**
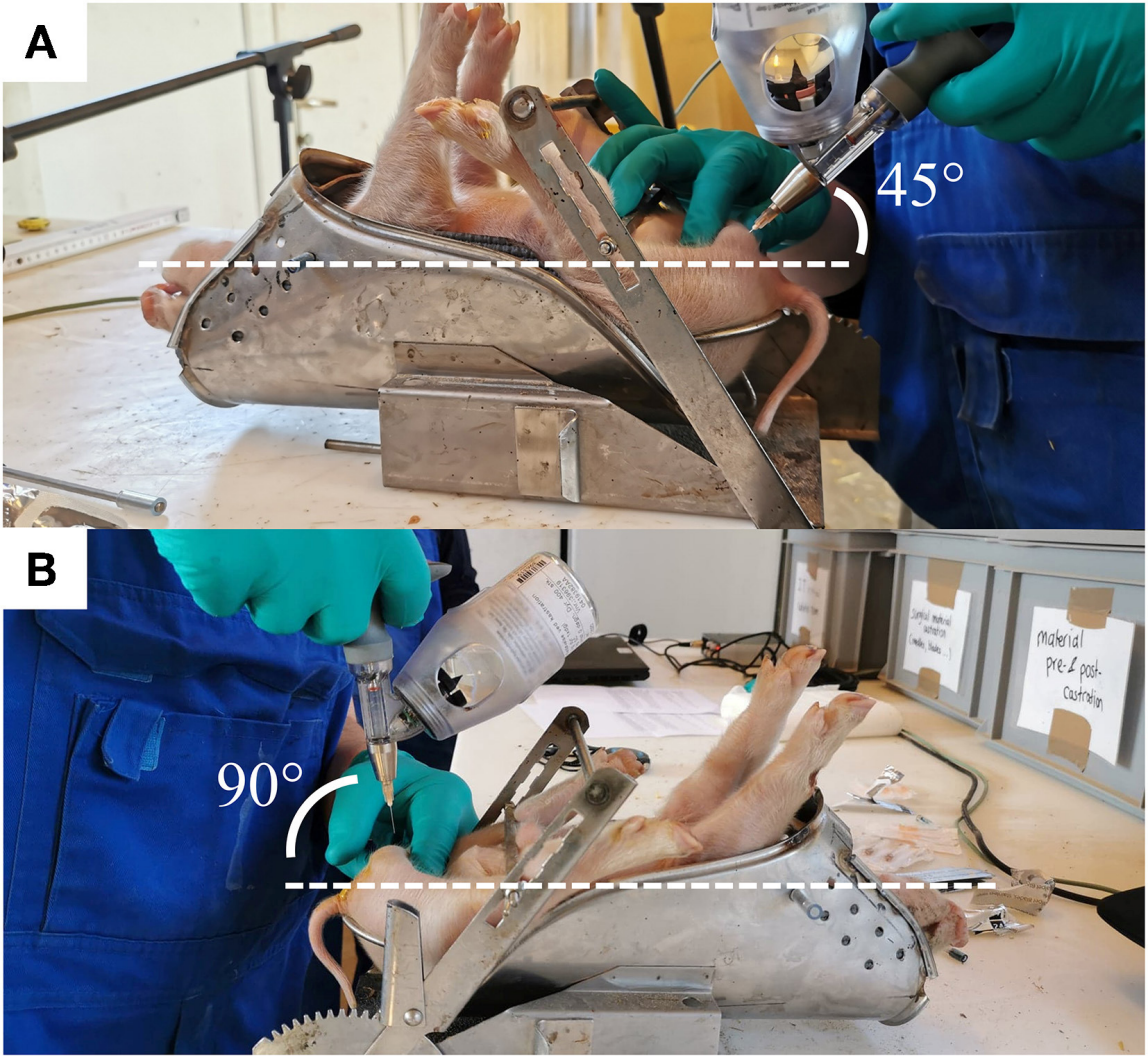
The two methods of injection of the local anesthetic. **(A)** The angle of insertion of the needle is shown for the intra-funicular (IF) and **(B)** intra-testicular (IT) methods.

Piglets were fixated in dorsal recumbency position in the castration bench, and testes were fixed carefully in the distal end of the scrotum. The scrotum area was not disinfected prior to anesthetic injection nor castration. The right testis was fixed caudally between the thumb and index finger of the experimenter, applying a steady but low pressure during the fixation. For the intra-funicular injection, the needle was inserted at a 45-degree angle pointing in dorsal direction and a 10-degree angle pointing in lateral direction from a caudocranial view (Figure 1). The needle was inserted in its full length (16 mm) through the center of the testis and aiming for the spermatic cord. The anesthetic was administered by continuously dispensing the drug while withdrawing the needle, and releasing the testis, also referred to as “push and pull technique” (19). After each injection, a drop of the anesthetic was left on the surface of the skin of the scrotum (*Cutis scroti)*. For the intra-testicular injection, the needle was inserted in the center of the right testis, in a dorsal direction at an angle of 90 degrees from a caudocranial view (Figure 1). A custom-made 5 mm plastic stopper was placed on each needle to ensure a standardized needle length of 7 mm. The anesthetic was injected slowly into the testicle (over approximately 3 s) while gradually loosening the grip around the testicle. The procedures were then repeated for the left testicle, and the piglet removed from the castration bench and placed in a heated area with littermates until castration.

#### Castration

After fixation in the castration bench, a disposable scalpel (Scalpel no. 24, carbon steel sterile blade, Swann-Morton, Sheffield, England) was used to perform an incision (approximately 1 cm) through the scrotal skin and spermatic fasciae. The right testis was then gently pressed between the index and the thumb of the experimenter until fully outside of the scrotum. The testis was then carefully lifted vertically, and the spermatic cord cut a few millimeters below the testis using the scalpel. The incision was repeated on the left testis. A new scalpel was used for each piglet. A video of the procedure is available in Supplementary Video S1.

#### Sham handling

Piglets were fixated in the castration bench, as previously described, for a duration of approximately 25 s (corresponding to the average duration of the procedures of local anesthesia and castration as assessed in a pilot study), during which they did not experience any tissue damage nor physical stimulation of the groin area.

### Ethical and other permits

The study was performed in compliance with the EU Directive 2010/63/EU for animal experiments, the Ministry of Food, Agriculture and Fisheries, and The Danish Veterinary and Food Administration under act 474 of 15. May 2014 and executive order 2028 of 14. December 2020. The experiment was approved as a clinical trial by the Danish Medical Agency (reference number 2020061784). All procedures were ethically evaluated and approved by the Danish Animal Experiments Inspectorate (Approval number 2019-15-0201-00263).

### Data collection

#### Vocalizations

The vocal responses of the piglets were recorded during each procedure, using a microphone (Sennheiser E614, Sennheiser Wennebostel, Germany) fixed 30 cm ahead of the piglet's snout, at the level of the head of the piglet. The microphone was connected to an amplifier (Audiobox USB^®^ 96, PreSonus, Louisiana, USA) connected to a computer, from which recordings were manually started and stopped upon piglet's placement and removal from the castration bench. Duration of each procedure was recorded. All vocal files were analyzed in Raven Pro 1.6 bioacoustics analysis software (Cornell Lab of Ornithology, Ithaca, New York, USA) using the band limited energy detector function, as described in Coutant et al. (27). This function allowed each intra-procedural call to be automatically detected based on a pre-set of parameters (data in Supplementary Text S1), and characterized in terms of number, duration, energy, and entropy. After running the automatic call detection, each procedural recording was manually checked to ensure that every call was properly selected, and to de-select surrounded noise or experimenter's voices wrongfully detected as a call. For all piglets, vocal characteristics of each procedure were then defined (Table 2) and analyzed. These procedures were performed by one person (MC), blinded to the experimental treatments. An auditory example of a piglet vocalizing during castration can be consulted in Supplementary Video S1.

**Table 2:** Description of the vocal parameters analyzed for each piglet during injection of local anesthetic, castration, or sham handling, all performed while the piglet was in the castration bench.

**Parameter (unit)**	**Description**
Call proportion	Proportion of time spent vocalizing during the procedure, calculated as call duration /procedure duration.
Call per second (s^−1^)	Number of calls per s of the procedure
Mean call duration (s)	Average duration of a call during the procedure, calculated as sum of call durations/number of calls.
Mean energy (dB)	Average energy, calculated as an average of the energy of each call during the procedure.
Max energy (dB)	Maximum value of energy recorded for all calls during the procedure.
Max power (dB)	The maximum power recorded for all calls during the procedure, relative to the specific recording set-up.
Aggregated entropy (kilobits)	Aggregated disorder for the procedure obtained by analyzing the energy distribution within each call. Higher entropy values correspond to greater disorder in the sound whereas a pure tone would have zero entropy (31).
Max entropy (kilobits)	Highest value of disorder recorded for all calls during the procedure.

#### Resistance movements

**Figure 2:**
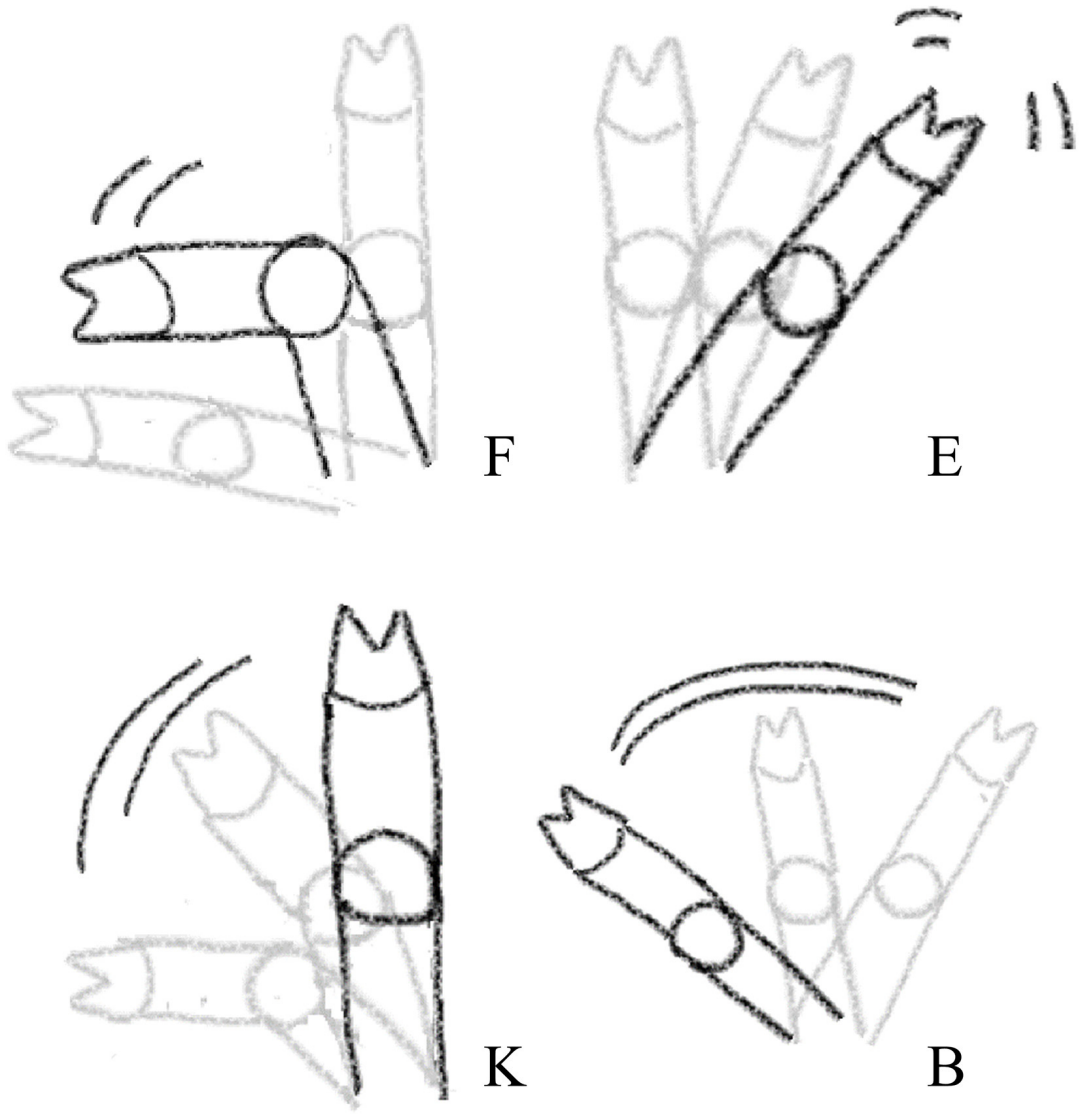
Visual representation of the four leg resistance movements recorded during injection of local anesthetic, castration, or sham handling while in the castration bench. F, Flexion; K, Kick; E, Extension; B, Blow. The visualizations only show the leg movements, see further description of the categories in Table 3.

Four distinct types of front leg movements, interpreted as resistance movements, were recorded during each procedure using a camera (GoPro HERO7 Black, GoPro, San Mateo, California, USA; 60 frames per sec, FPS) placed on a stand 30 cm to the right of the castration bench, approximately 50 cm above the bench. This distance allowed a full picture of the piglets' front legs. Resistance movements were quantified using a novel method developed in Coutant et al. (27). Video clips were observed at low speed (5 FPS) to detect four types of movements: flexion, extension, kick, and blow (Table 3; Figure 2). Two observers, blinded to the experimental treatments, were trained to recognize and count these behaviors, and practiced recording on approximately 100 random video clips, using the Behavioral Observation Research Interactive Software [BORIS; (32)]. Each video sequence was then analyzed, and the occurrence of each type of behavior was counted for each front leg in the interval between closing and opening of the castration bench. Movements that were too sudden to be categorized despite the low speed of video analysis were not counted. Reversely, movements performed relatively slow (duration >1 s) were not considered as resistance and therefore not recorded. In addition, duration of blocking in the bench, corresponding to a leg being mechanically unable to move due to physical blocking, were also recorded. The two observers showed a high inter-observer reliability with an intraclass correlation coefficient (ICC) close to 96% (95% confidence interval: 90–99%; comparing 15 video clips). An example of leg resistance movements performed by a piglet during castration can be consulted in the video in Supplementary Video S1.

**Table 3:** Description of the leg resistance movements recorded during injection of local anesthetic, castration, or sham handling, all performed while the piglet was in the castration bench.

**Category**	**Description**
Flexion	Piglet vertically bends his front leg, provoking a flexion of the elbow of at least 90 degrees.
Extension	Piglet fully extends his front leg while lowering the head in the bench. May be accompanied by trembling of the leg and/or by a subtle lift of the piglet's back.
Kick	Piglet front leg performs a sudden upwards movement, changing from a flexion to a tense upwards position.
Blow	Piglet suddenly draws back his front leg forwards or backwards for at least half a bench length, from a normal upright position to an extended position, with little or no flexion of the elbow.
Leg blocked	Piglet's front leg is blocked in the bench cone, preventing movement.

Each leg was scored separately. Figure 2 shows a visualization of the leg movements.

#### Saliva cortisol concentrations

For baseline, one saliva sample per piglet was collected in the home pen on average 35 (±14; SD) min before the first procedure. For changes in saliva cortisol in response to the procedures, one sample per piglet was collected on average 17 (±9) min after castration and again approximately 6 h after castration (5 h 47 min ±26 min). Saliva samples were performed using a novel method recently used in Coutant et al. (27); a cotton swab (Salivette^®^, Sarstedt, Aktiengesellschaft & Co., Numbrecht, Germany) was cut in pieces (approx. 2.0 × 0.5 cm), soaked in concentrated apple juice (nectar from concentrated juice, min 60%, Rynkeby Foods A/S, Ringe, Denmark) for 1 h, and dried in an electric oven at 60°C for 5 h. A pilot study revealed an increase in saliva production with this method compared to the use of a non-pre-soaked piece of cotton. Similar results were obtained with soaking the cotton pieces in citric acid (fresh lemon juice), but after this method, saliva sampling seemed more aversive for the piglets, and the method was therefore abandoned. During sampling, the cotton swab was fixed at the end of a straight pean clamp, and gently introduced into the piglet's mouth, while the piglet was held in the experimenter's arms. The cotton was rotated gently in the piglet's mouth for 30 to 45 s, with insistence around the salivary glands. This procedure was performed by one of four trained experimenters blinded to the experimental treatments. The sample was then placed in an experimental tube (provided as part of the Salivette^®^), labeled, and stored at −18°C until cortisol concentration determination at the departmental laboratory. Samples were defrosted and centrifuged for 6 min at 1,000 × *g*. Concentrations of cortisol were determined using a direct enzyme immunoassay without extraction and previously validated for saliva (Arbor Assays, Cat. K003-H1W, Michigan, USA). With this method, the antiserum cross-reacts with cortisol and some cortisol metabolites, and values have to be interpreted as cortisol immunoreactivity. The intra-assay coefficient of variation was 3.7 and 5.6%, respectively, for low and high control, and the inter-assay variation was 7.2 and 9.8% for low and high control, respectively. The minimal detectable concentration was 45 pg/mL. The procedure outlined by the manufacturer was followed.

### Statistical analysis

Three piglets were removed from the analysis: two piglets due to experimental issues during the procedures, and one due to cryptorchidism (only one testis descended) discovered during injection of the local anesthetic. In addition, for two piglets, insufficient amounts of saliva rendered cortisol analysis impossible, and eight piglets were excluded from the resistance movement analysis due to technical issues with the recording of the videos (five for injection and three for castration). Malfunctioning and technical issues of the vocalization recording set-up resulted in 168 missing files for anesthesia and 182 missing files for castration, leaving valid vocalization data from 376 piglets for anesthesia and 412 piglets for castration.

Vocalizations and resistance movements were analyzed separately for local anesthesia and castration. During the injection of the local anesthetic, time interval had not yet any bearing, and thus for the first procedure only three treatments were relevant: injection by intra-funicular method (IF), injection by intra-testicular method (IT), and sham handling (SH).

The vocalization parameters (Table 2) were log-transformed if necessary (to obtain normality) and analyzed in a linear mixed effects model with treatment as the main explanatory variable, weight (range: 0.94–2.68 kg), age (3 or 4 days), time of day when starting the procedure (decimal h, range: 8.21–14.66) and duration of procedure (range: 21–117 s) as covariates, and litter as a random effect. Rate of vocalizations and other variables where duration of the procedure was an integrated part of the calculated response, were analyzed by similar models, but without duration of the procedure as covariate.

The counts of each type of resistance movements were summed per piglet during each procedure (Table 3). Total observation duration was defined as the sum of observation time per leg, subtracting the duration of left leg and right leg occasionally being blocked while in the castration bench. The sum of piglets' resistance movements was analyzed by a negative binomial mixed effects model including treatment as main explanatory variable, weight, age and time of day as covariates, logarithm of total observation duration (range: 1–211 s) as offset, and litter as a random effect.

Changes in saliva cortisol in response to the procedures were log transformed and analyzed in a mixed model with treatment, sampling point (early - late) and their interaction as fixed effects of main interest, and weight, age, time of sampling (decimal h, range: 7.82–20.50), and baseline cortisol concentration (range: 2,001–48,570 pg/mL) as covariates, with sample point as a repeated measure, and litter as random effect.

For all outcomes, initial models were reduced by stepwise removal of fixed effects at *P* > 0.10, starting with the interaction of highest order, however, never removing the main effect for the variables of key interest. In linear mixed effects models, Satterthwaite's approximation of denominator degrees of freedom was used. Deviations from assumption of normality and variance homogeneity were monitored visually by plotting residuals at each step. Covariates with significant effects were maintained in the final models, but effects not reported, at the exception of weight.

In case the final model showed significant effect of the treatments (*P* ≤ 0.05), pairwise comparisons between treatments were performed with *p*-values adjusted for multiple comparisons using the Tukey-Kramer method (marked by P_adj_). For analyses of castration, however, Tukey-Kramer was not applied as the 13 treatment groups would result in 78 pairwise comparisons, many of which were not relevant for the aims of the present study. Instead, intervals were compared within injection methods, and methods were compared within intervals, using unadjusted *P*-values reported as *P*. All calculations were performed using SAS 9.4 (SAS Institute Inc., Cary, North Carolina, USA). All data used for statistical analysis can be consulted in the dataset in Supplementary Dataset S1. Descriptive measures are presented as average ± standard error.

## Results

### Vocalizations

Vocal responses of piglets during administration of the local anesthetic were affected by the treatments. A significant difference was observed in 6 out of 8 indicators (Table 4), i.e., for call proportion, call per second, mean energy, max energy, max power, and aggregated entropy. Across all indicators, no differences were found between the two methods of injection of the local anesthetic, and both types of procedures resulted in higher values of vocal parameters compared to sham handled (i.e., not injected, not castrated) piglets. There was no effect of treatments in terms of mean call duration and max entropy.

**Table 4:** Averages (± SE) of vocal parameters recorded during injection of the local anesthetic.

	**IF**	**IT**	**SH**	**F-test**	***P***
Call proportion	0.63 ± 0.01*^*a*^*	0.64 ± 0.01*^*a*^*	0.47 ± 0.02*^*b*^*	F_2, 314 =_ 29.4	< 0.001
Call per second (s^−1^)	0.90 ± 0.02*^*a*^*	0.88 ± 0.02*^*a*^*	0.66 ± 0.03*^*b*^*	F_2, 314 =_ 39.3	< 0.001
Mean call duration (s)	0.75 ± 0.03	0.76 ± 0.02	0.72 ± 0.03	F_2, 311 =_ 1.6	0.201
Mean energy (dB)	84.80 ± 9.17*^*a*^*	85.13 ± 9.12*^*a*^*	46.41 ± 10.10*^*b*^*	F_2, 310 =_ 5.6	0.004
Max energy (dB)	319.98 ± 8.54*^*a*^*	318.51 ± 7.94*^*a*^*	288.21 ± 10.46*^*b*^*	F_2, 311 =_ 3.2	0.041
Max power (dB)	−14.24 ± 0.74*^*a*^*	−14.40 ± 0.67*^*a*^*	−17.06 ± 0.89*^*b*^*	F_2, 311 =_ 4.3	0.015
Agg entropy (kilobits)	130.54 ± 3.27*^*a*^*	122.08 ± 2.85*^*a*^*	108.82 ± 4.40*^*b*^*	F_2, 330 =_ 33.8	< 0.001
Max entropy (kilobits)	5.94 ± 0.01	5.91 ± 0.01	5.93 ± 0.02	F_2, 312 =_ 1.1	0.340

^*a, b*^Different letters within a row indicate significant differences between treatments, P_*adj*_ ≤ 0.05; IF, intra-funicular injection (n = 138); IT, intra-testicular injection (n = 135); SH, sham anesthesia (n = 103). F_*n, d*_ denotes an F-test on n, numerator and d, denominator degrees of freedom for the effect of treatment.

At castration, all vocalization indicators showed significant treatment differences (Table 5), except for max entropy. Generally, IF did not differ significantly from IT, except in mean call duration, where IF resulted in longer calls than IT when pigs were castrated 2.5 min after anesthetic injection, while IT resulted in longer calls than IF when pigs were castrated after 30 min. Piglets castrated 2.5 min after IF injection showed mean call durations, maximum energy and maximum power levels not differing significantly from CC. Additionally, no differences were found between vocal responses of piglets castrated 30 min after IT injections and the ones of CC in terms of call proportion, mean call duration and max energy. In five out of eight indicators (i.e., call proportion, mean call duration, mean energy, max energy, and max power), no differences were found between vocal responses of IT and IF vs. SH, when piglets were castrated after 5 and 10 min. Vocal responses of piglets in the treatments IF and IT were, though, greater than SH in the case of castration after 2.5 and 30 min. IT and IF vocal responses were greater than SH, regardless of the interval between local anesthesia and castration, and did not differ significantly from those of CC, in terms of call per second and aggregated entropy. Piglet weight was associated with mean energy (F_1, 289 =_ 7.1, *P* = 0.008), max energy (F_1, 281 =_ 4.6, *P* = 0.033), and max power parameters (F_1, 277 =_ 7.5, *P* = 0.007), with greater values observed in heavier pigs. Overall, piglets' vocal responses to castration did not differ between intra-testicular and intra-funicular injections. Stronger responses, often comparable to those of piglets castrated without anesthesia, were shown by piglets castrated 2.5 and 30 min after anesthetic injection, while most vocal parameters did not differ from sham handled animals when piglets were castrated 5 and 10 min after local anesthesia. For two indicators, i.e., call rate and aggregated entropy, the vocal parameters of piglets castrated after injection of local anesthetic did not differ significantly from those of piglets castrated without anesthesia, regardless of the time interval between local anesthesia and castration.

**Table 5:** Averages (±SE) of vocal parameters recorded during castration.

		**IF**	**IT**	**SH**	**F-test**	***P***
Call proportion	2.5 min	0.61 ± 0.03*^*b*, 1^*	0.59 ± 0.03*^*b*, 1^*	0.43 ± 0.04*^*b*, 2^*	F_12, 370_ = 6.8	< 0.001
	5 min	0.59 ± 0.03*^*b*^*	0.60 ± 0.03*^*b*^*	0.51 ± 0.07*^*b*^*		
	10 min	0.55 ± 0.03*^*b*, 1^*	0.58 ± 0.02*^*b*, 1/2^*	0.44 ± 0.05*^*b*, 2^*		
	30 min	0.60 ± 0.03*^*b*, 1^*	0.66 ± 0.03*^*ab*, 1^*	0.43 ± 0.04*^*b*, 2^*		
	CC	0.72 ± 0.02*^*a*^*	0.72 ± 0.02*^*a*^*	0.72 ± 0.02*^*a*^*		
Call per second	2.5 min	0.87 ± 0.05*^1^*	0.87 ± 0.05*^1^*	0.68 ± 0.05*^*b*, 2^*	F_12, 363_ = 6.7	< 0.001
	5 min	0.91 ± 0.04*^1^*	0.89 ± 0.05*^1^*	0.63 ± 0.07*^*b*, 2^*		
	10 min	0.95 ± 0.04*^1^*	0.94 ± 0.03*^1^*	0.69 ± 0.07*^*b*, 2^*		
	30 min	0.94 ± 0.04*^1^*	0.87 ± 0.04*^1^*	0.64 ± 0.05*^*b*, 2^*		
	CC	0.92 ± 0.04	0.92 ± 0.04	0.92 ± 0.04*^*a*^*		
Mean call duration (s)	2.5 min	0.75 ± 0.05*^*ab*, 1^*	0.70 ± 0.05*^*abc*, 2^*	0.63 ± 0.04*^*b*, 2^*	F_12, 364_ = 2.4	0.006
	5 min	0.70 ± 0.05*^*bc*^*	0.70 ± 0.05*^*b*^*	0.81 ± 0.11*^*b*^*		
	10 min	0.60 ± 0.04*^*c*^*	0.62 ± 0.03*^*c*^*	0.67 ± 0.08*^*b*^*		
	30 min	0.68 ± 0.04*^*bc*, 2^*	0.81 ± 0.05*^*ab*, 1^*	0.68 ± 0.05*^*b*, 2^*		
	CC	0.82 ± 0.04*^*a*^*	0.82 ± 0.04*^*a*^*	0.82 ± 0.04*^*a*^*		
Mean energy (dB)	2.5 min	34.58 ± 15.75*^*b*, 1^*	52.37 ± 16.6*^*b*, 1^*	0.15 ± 15.52*^*b*, 2^*	F_12, 358_ = 4.8	< 0.001
	5 min	25.88 ± 16.43*^*b*^*	29.59 ± 18.05*^*b*^*	42.88 ± 29.40*^*b*^*		
	10 min	19.28 ± 19.23*^*b*^*	12.93 ± 11.93*^*b*^*	−2.34 ± 21.16*^*b*^*		
	30 min	50.27 ± 18.81*^*b*, 1^*	59.10 ± 18.68*^*b*, 1^*	6.74 ± 15.40*^*b*, 2^*		
	CC	131.20 ± 16.32*^*a*^*	131.20 ± 16.32*^*a*^*	131.20 ± 16.32*^*a*^*		
Max energy (dB)	2.5 min	299.20 ± 16.71*^*ab*, 1^*	302.53 ± 15.36*^*b*, 1^*	244.89 ± 18.90*^*b*, 2^*	F_12, 359_ = 3.5	< 0.001
	5 min	273.55 ± 18.78*^*bc*^*	285.58 ± 19.65*^*b*^*	271.39 ± 24.91*^*b*^*		
	10 min	265.70 ± 20.45*^*c*^*	268.42 ± 17.46*^*b*^*	250.20 ± 26.68*^*b*^*		
	30 min	292.07 ± 18.28*^*bc*, 1^*	302.51 ± 14.16*^*ab*, 1^*	243.05 ± 18.59*^*b*, 2^*		
	CC	349.24 ± 11.32*^*a*^*	349.24 ± 11.32*^*a*^*	349.24 ± 11.32*^*a*^*		
Max power (dB)	2.5 min	−15.22 ± 1.52*^*ab*, 1^*	−15.20 ± 1.34*^*b*, 1/2^*	−20.20 ± 1.79*^*b*, 2^*	F_12, 359_ = 3.2	0.002
	5 min	−18.42 ± 1.73*^*c*^*	−17.44 ± 1.73*^*b*^*	−18.65 ± 2.02*^*b*^*		
	10 min	−17.87 ± 1.64*^*bc*^*	−19.36 ± 1.59*^*b*^*	−20.52 ± 2.20*^*b*^*		
	30 min	−15.92 ± 1.54*^*bc*, 1^*	−15.92 ± 1.15*^*b*, 1^*	−20.39 ± 1.54*^*b*, 2^*		
	CC	−11.83 ± 1.04*^*a*^*	−11.83 ± 1.04*^*a*^*	−11.83 ± 1.04*^*a*^*		
Agg. entropy (kilobits)	2.5 min	136.12 ± 7.23*^1^*	136.52 ± 10.25*^1^*	110.08 ± 9.03*^*b*, 2^*	F_12, 363_ = 6.3	0.001
	5 min	144.72 ± 12.55*^1^*	138.54 ± 7.39*^1^*	100.73 ± 11.52*^*b*, 2^*		
	10 min	149.04 ± 7.25*^1^*	141.56 ± 6.84*^1^*	103.88 ± 9.52*^*b*, 2^*		
	30 min	149.11 ± 7.48*^1^*	133.58 ± 5.92*^1^*	108.61 ± 9.11*^*b*, 2^*		
	CC	148.54 ± 6.23	148.54 ± 6.23	148.54 ± 6.23*^*a*^*		
Max entropy (kilobits)	2.5 min	5.97 ± 0.03	5.96 ± 0.03	5.93 ± 0.02	F_12, 361_ = 1.7	0.067
	5 min	6.00 ± 0.03	6.00 ± 0.03	5.97 ± 0.03		
	10 min	5.99 ± 0.04	5.99 ± 0.04	5.98 ± 0.04		
	30 min	5.96 ± 0.03	5.98 ± 0.03	6.00 ± 0.02		
	CC	5.89 ± 0.03	5.89 ± 0.03	5.89 ± 0.03		

^1, 2^Different numbers within a row indicate significant differences between injection methods within intervals for the given parameter. ^*a*−*c*^Different letters within a column indicate significant differences between interval within injection method fort the given parameter; IF, intra-funicular injection and castration after 2.5 min (n = 35), 5 min (n = 33), 10 min (n = 33) or 30 min (n = 36); IT, intra-testicular injection after 2.5 min (n = 30), 5 min (n = 35), 10 min (n = 36) or 30 min (n = 35); SH, sham handling at an interval of 2.5 min (n = 35), 5 min (n = 16), 10 min (n = 19) or 30 min (n = 35); CC, castration without pain mitigation (control-castrated, n = 35). F_*n, d*_ denotes an F-test on n, numerator and d, denominator degrees of freedom for the effect of treatment.

### Resistance movements

Piglets' leg movements during injection of the local anesthetic differed significantly among treatments, with greater levels of movements observed in IF and IT than in SH. The leg movements observed during intra-testicular and intra-funicular injections did not differ significantly, although piglets injected by the intra-funicular method displayed an average of 17.6% more leg movements than piglets injected by the intra-testicular method. At castration, the number of resistance movements differed significantly among treatments (Table 6). Regardless of the time interval between injection and castration, IF did not lead to a significantly different response from IT, and both methods resulted in more resistance movements than SH. Piglets castrated at various intervals after IF injection did not differ significantly in their response, but piglets castrated after 30 min showed a response that did not differ significantly from the one of CC. Similarly, piglets castrated after IT injection did not differ significantly in their response, regardless of the interval. In addition, levels of resistance movements of piglets castrated after 2.5 or 30 min did not differ significantly from the ones of CC. All SH piglets responded significantly less than castrated piglets, regardless of interval between stays in the castration bench. Overall, the level of resistance movements during castration did not differ between injection methods nor interval between procedures. Responses of piglets castrated 2.5 or 30 min after injection of anesthetic led to responses that did not differ from those of piglets castrated without anesthesia. In addition, anesthetised piglets, regardless of the method, showed more resistance movements than sham handled piglets.

**Table 6:** Averages (±SE) of leg resistance movements recorded during castration.

		**IF**	**IT**	**SH**	**χ^2^-test**	***P***
**Anaest**.		28.44 ± 1.26*^*a*^*	23.85 ± 1.02*^*a*^*	14.32 ± 1.08*^*b*^*	$X_{2}^{2}$ = 172.4	< 0.001
**Cast**.	**2.5 min**	24.62 ± 2.13*^*b*, 1^*	30.26 ± 2.71*^*ab*, 1^*	13.96 ± 1.77*^*b*, 2^*		< 0.001
	**5 min**	24.23 ± 2.58*^*b*, 1^*	26.49 ± 2.63*^*b*, 1^*	11.08 ± 2.58*^*b*, 2^*		
	**10 min**	30.22 ± 3.85*^*b*, 1^*	26.96 ± 2.65*^*b*, 1^*	16.08 ± 3.34*^*b*, 2^*	$X_{2}^{2}$ = 132.4	
	**30 min**	29.51 ± 2.41*^*ab*, 1^*	30.96 ± 3.00*^*ab*, 1^*	15.69 ± 2.01*^*b*, 2^*		
	**CC**	41.06 ± 2.72*^*a*^*	41.06 ± 2.72*^*a*^*	41.06 ± 2.72*^*a*^*		

At anesthesia (Anaest.), ^*a, b*^different letters within a row indicate significant differences between treatments, P_*adj*_ ≤ 0.05; IF, intra-funicular injection (n = 195); IT, intra-testicular injection (n = 196); SH, sham anesthesia (n = 148). At castration (Cast.), ^1.2^ different numbers within a row indicate significant differences between methods within intervals, P ≤ 0.05. ^*a*−*c*^Different letters within a column indicate significant differences between intervals within methods, P ≤ 0.05. IF, intra-funicular injection and castration after 2.5 min (n = 50), 5 min (n = 48), 10 min (n = 50) or 30 min (n = 49); IT, intra-testicular injection after 2.5 min (n = 50), 5 min (n = 49), 10 min (n = 49) or 30 min (n = 49); SH, sham handling at an interval of 2.5 min (n = 50), 5 min (n = 25), 10 min (n = 25) or 30 min (n = 48); CC, castration without pain mitigation (control-castrated, n = 49). $\chi_{n}^{2}$ denotes a chi-squared test on n degrees of freedom for the effect of treatment.

### Saliva cortisol concentrations

As expected, baseline cortisol concentrations from samples obtained 35–40 min before the procedures did not differ significantly among treatments (approx. 9,700 pg/mL, F_12, 503 =_ 0.8, *P* = 0.673; Table in Supplementary Table S1). A weight effect was observed (F_1, 485 =_ 12.2, *P* < 0.001), with higher values of baseline cortisol in lighter piglets.

The interaction between treatments and timing of sampling was not significant (F_12, 1044 =_ 1.56, *P* = 0.097). After removing the interaction from the model, the main effect of treatment was also not significant, although a tendency was observed (F_12, 1112_ = 1.67, *P* = 0.067). However, the average cortisol concentrations following the procedures were significantly affected by the sample point (F_1, 1056_ = 708.3, *P* < 0.001), with higher concentrations observed 17 min after castration (17,378 ± 8,216 pg/mL) than 6 h after castration (9,479 ± 5,672 pg/mL). In this model, an effect of weight was also observed (F_1, 456 =_ 6.2, *P* = 0.013), with higher cortisol concentration in lighter piglets.

Considering the significant difference between cortisol concentrations measured at 17 min and 6 h post-procedure, two separate analyses were performed. At 17 min, treatment groups differed in cortisol concentration (Table 7). Across IF and IT, no difference was found among intervals between injection and castration, but both injection methods led to, or tended to lead to, a greater response than shown by sham piglets at 10 and 30 min. Within the IF treatments, piglets showed a greater cortisol response when castrated 30 min after administration of the local anesthetic compared to 2.5 and 5 min. Piglets anesthetised by the IT method did not differ significantly, irrespective of the time interval between injection of the local anesthetic and castration. Regardless of the method of injection of the local anesthetic, piglets castrated after 10 and 30 min showed a greater cortisol response than CC. Within SH, no differences were observed for intervals 2.5, 5, and 30 min, but piglets offered a 10 min interval between stays in the castration bench had a lower cortisol concentration compared to intervals of 2.5 min and 5 min. The latter intervals were also significantly different from CC. Cortisol responses decreased with piglet weight (F_1, 356_ = 9.49, *P* = 0.002). Overall, cortisol responses 17 min after castration did not differ between injection methods, but greater responses were observed in piglets castrated 10 or 30 min after injection, compared to piglets castrated after 2.5 or 5 min, and to piglets castrated without anesthesia.

**Table 7:** Averages (±SE) of saliva cortisol concentrations (pg/mL) sampled at 17 min post-castration.

	**IF**	**IT**	**SH**	**F-test**	***P***
2.5 min	16,958 ± 1,100*^*bc*^*	17,518 ± 1,277*^*ab*^*	18,589 ± 1,255*^*a*^*	F_12, 518 =_ 2.5	0.003
5 min	16,367 ± 1,196*^*bc*^*	16,291 ± 816*^*ab*^*	18,742 ± 1,750*^*ab*^*		
10 min	19,132 ± 1,289*^*ab*, 1^*	19,605 ± 1,482*^*a*, 1^*	13,687 ± 1,289*^*c*, 2^*		
30 min	19,713 ± 1,325*^*a*, 1^*	17,207 ± 936*^*a*, 1/2^*	15,842 ± 1,053*^*ab*, 2^*		
CC	15,057 ± 858*^*c*^*	15,057 ± 858*^*b*^*	15,057 ± 858*^*b*^*		

^1, 2^Different numbers within a row indicate significant differences between methods within intervals. ^*a, b*^Different letters within a column indicate significant differences between intervals within methods, P ≤ 0.05. IF, intra-funicular injection and castration after 2.5 min (n = 50), 5 min (n = 48), 10 min (n = 50) or 30 min (n = 49); IT, intra-testicular injection after 2.5 min (n = 50), 5 min (n = 48), 10 min (n = 49) or 30 min (n = 49); SH, sham handling at an interval of 2.5 min (n = 50), 5 min (n = 25), 10 min (n = 25) or 30 min (n = 49); CC, castration without pain mitigation (control-castrated, n = 49). F_*n, d*_ denotes an F-test on n numerator and d denominator degrees of freedom for the effect of treatment.

At 6 h post-procedure, piglets' cortisol responses did not differ significantly among treatments (F_12, 523 =_ 0.7, P=0.721, Table in Supplementary Table S1). At this point in time, cortisol concentrations were not associated with piglet weight (F_1, 301 =_ 0.2, *P* = 0.681).

## Discussion

We aimed in this study to assess piglets' acute responses to two methods of injection of a local anesthetic, and surgical castration following four different time intervals after the injection. Acute responses were evaluated based on piglet vocalizations, leg movements interpreted as resistance, and saliva cortisol concentrations in samples obtained at two time points after castration (at approximately 17 min and approximately 6 h). Results showed no significant difference between the two injection methods. Greater acute responses were observed during castration performed 2.5 and 30 min compared to 5 or 10 min after the injections. Saliva cortisol concentrations in samples obtained from piglets castrated after injection with the local anesthetic did not differ significantly from those of piglets castrated without any anesthesia or sham handled. Below, these findings are discussed in terms of methodology and in relation to animal welfare.

Our results showed that, overall, administration of the procaine-based local anesthetic reduced the acute responses of piglets to castration, as measured by the number of foreleg movements interpreted as resistance and vocalization characteristics (including number, duration, and intensity of calls), as compared to piglets castrated without anesthesia. These results are based on quantitative recording of resistance movements and automatic detection and characterization of vocal parameters developed in the study, and are in line with previous results (11, 14, 23, 27). Thus, these methods seem to be able to detect subtle differences in piglets' acute responses to early-life interventions.

Saliva-sampling of 3–4 day old piglets is difficult as they produce relatively low amounts of saliva, and display less spontaneous chewing on cotton swabs during sampling than older piglets being experienced with solid feed intake. The present technique, developed in Coutant et al. (27), showed successful as it allowed gathering of enough saliva to perform the assays. In addition, samples taken on average 17 min after castration showed significantly higher cortisol concentrations than baseline samples taken before, and post-procedural samples taken hours after the intervention, indicating that these samples did record a robust response to the procedure.

The present findings on saliva cortisol are, however, not in line with previous studies showing a lowered cortisol response to castration in piglets administered a local anesthetic prior to castration compared to piglets castrated without anesthesia (10, 15, 33). Importantly, previous studies reporting these results analyzed plasma cortisol, and used lidocaine as the anesthetic, potentially in combination with an analgesic. To the best of our knowledge, only one other study reported saliva cortisol responses to castration following injection of procaine (27). This study, using a similar design to the present one, showed no difference in cortisol response between piglets castrated with and without anesthesia. A study on the development of the circadian pattern of saliva cortisol secretion in neonatal piglets reported a relatively high variation in cortisol concentrations from saliva sampled in piglets up to 3 days of age, with a stable circadian pattern only observed from 10 days of age in males (34). With a relatively large individual variation in cortisol response, even within a treatment, it cannot be excluded that the variation in saliva cortisol concentrations in piglets as young as 3 to 4 days of age may have reduced the possibility to detect differences in acute responses, despite our relatively large sample size (calculated based on previous plasma cortisol results). Further studies investigating the plasma cortisol response of piglets subjected to treatments similar to our study could therefore be relevant, although the general impact of the sampling method should be carefully evaluated. We suggest that this way of saliva sampling is generally preferable as relatively non-invasive compared to venepuncture.

In relation to the interpretation of the cortisol results, concern has been raised for years regarding the usefulness of cortisol and other physiological indicators to inform about the affective component of pain, as these indicators may be more related to the level of arousal induced by the procedure (35, 36). Our results may therefore also reflect piglets' stress response following the combined procedures rather than the ability of the anesthetic to relieve acute pain only. If a longer duration of the procedure (including injection of anesthetic, castration, and the time interval in-between) is correlated with higher stress responses, this suggestion could explain why, in our study, higher cortisol responses were observed in piglets castrated 10 or 30 min after administration of the local anesthetic, compared to 2.5 or 5 min after, and compared to piglets non-anesthetized. It is, however, surprising that a similar pattern was not observed in sham handled piglets, where a 10 min interval between stays in the castration bench led to a lower cortisol response than 2.5 or 5 min intervals. It is possible that a longer interval between stays in the bench resulted in a different shape of the curve of cortisol response, the peak of which is not known. We also hypothesize that the lack of sensitivity of cortisol may have resulted in ceiling effects following handling alone, as already suggested by previous authors (4, 11), especially in neonatal piglets, whose HPA-axis may be highly responsive (33). This suggestion is supported, in our study, by comparable cortisol levels recorded in sham handled and castrated piglets. As a consequence of these known concerns, the present study examined acute responses to injection of anesthetic and castration across several indicators including cortisol, vocalizations and resistance movements, thereby taking a multi-modal approach as suggested by Sheil and Polkinghorne, and Baysinger et al. (36, 37).

To the best of our knowledge, only one other study has investigated the efficacy of a local anesthetic as pain mitigating when administered in practice by farmers. The authors concluded that herdsmen were able to effectively inject a local anesthetic intra-testicularly, resulting in comparable efficacy of anesthesia as reported in other studies involving trained veterinarians (13). Yet, this assessment was concluded with no data or consideration of the piglets' response to the injection of the local anesthetic itself. Our study was not designed to assess herdsmen's ability to administer the local anesthetic and subsequently perform castration, but rather to study the acute response of the piglets, with approximation to on-farm practice. A few adaptations to the castration routine of the commercial farm had to be implemented for the sake of data recording and standardization though. Piglets were brought outside of the farrowing room to be injected with the local anesthetic and castrated while in a castration bench, whereas many farmers perform castration in the farrowing room, placing the piglets upside down or onto the herdsman's lap during both procedures. Although the impact of castration performed while the piglet is held upside down has recently been reported (38), comparison of the potential implications of different restraining techniques for piglets' responses to injection of local anesthetics has not yet been investigated. We cannot exclude that fixation in the castration bench led to a different stress response compared to handling on the lap, however, we would expect that the placement of piglets in a calm, heated area with littermates between injection and castration contributed to limiting the stress response compared to the typical on-farm practice of placement in a cartwheel in the farrowing room. Similarly, application of the procedures in a calm environment may limit the emotional social contagion of stress responses (among littermates, and with the sow) potentially happening when several litters are processed simultaneously in the farrowing room (39). Yet, further studies are required to investigate these suggestions. Throughout the study, the same trained herdsman, hired for this specific role, performed the procedures on all experimental piglets. This was chosen to strengthen accuracy, lower variation and thus increase the power to detect differences when comparing experimental treatments. Whether the results can translate directly into a farm setting is however not known. Further studies could investigate the efficacy of the administration of a local anesthetic when performed by farm employees, as part of their daily routines in the farrowing room.

Our study did not show a significant difference in acute responses of piglets exposed to the intra-testicular vs. intra-funicular injection methods. This result is in line with a previous study performed in piglets under general anesthesia, and also comparing the two methods (40). It can be remarked, though, that the study by Haga & Ranheim (40) included a lidocaine-based drug, and involved piglets of 22 days of age, thus significantly older than in the present study. Similarly, a recent study found no difference in vocalization and leg movements in piglets administered 0.5 mL of procaine by an intra-funicular injection, vs. 0.3 mL of the drug administered by intra-testicular injection (23). The latter study involved the administration of different volumes of anesthetic for the two injection methods, evaluating the combined effects of the intra-testicular pressure resulting from the volume of liquid injected (20), the anesthetic efficacy obtained from the dose of anesthetic administered, and the pain response from the area of injection (21). Although the present results did not reach statistical significance, our data showed close to 20% more resistance movements during injection of the anesthetic in piglets subjected to an intra-funicular injection vs. intra-testicular injection. The intra-funicular injection is quite complex, requiring a deeper, more precise, injection into the testicle and toward the spermatic cord. Precision of the injection is considered important for the efficacy of the anesthetic (41), while the speed of the drug injection and the intra-testicular pressure may affect pain from the injection (21). If one of the injection methods is to be applied, we therefore see an advantage of the use of the intra-testicular injection, as it appears to be easier and faster to perform than the currently used intra-funicular injection type (23), with comparable efficacy at castration. Yet, penetration of the testicle by the needle, and increased intra-testicular pressure resulting from the intra-testicular injection are considered painful (41). In our study, injection of the local anesthetic, regardless of the method of injection, led to a rate of resistance movements close to doubled compared to handling without injection. In addition, the duration of the procedure was affected by piglet weight (results not shown), with longer procedures observed in lighter piglets, potentially because fixation of the testes and insertion of the needle at a correct angle may have been more difficult in small piglets. Weight and age effects were also observed in the vocal responses of the piglets to the administration of the anesthetic and to castration, and in the saliva cortisol responses as well. These effects may be related to a different fit in the castration bench for smaller piglets, and/or to weight-related characteristics, such as a smaller thoracic cage, potentially affecting the performance of vocalizations. It is, however, also possible that piglets' experience of anesthesia and castration vary based on their weight, as the volume of anesthetic injected was fixed, and the dose of anesthetic administered per kg therefore decreased with an increase in weight. This possibility is, however, poorly supported by our data as weight effects were inconsistent and not recurrent across indicators. Yet, future studies could focus on the effects of anesthetic injection and subsequent castration applied in piglets across a wider weight range, e.g., across the legal age range of 2 to 7 days for surgical castration after local anesthesia.

The effect of interval between injection of local anesthetic and castration is poorly investigated in the literature, and seem to only have been included in one study by Courboulay et al. (30). The study compared piglets' response to castration 15 or 30 min following administration of procaine, and found reduced plasma cortisol response in piglets castrated 30 min compared to 15 min after injection (30). The interpretation of this result in terms of the efficacy of the anesthetic can be questioned, though, as cortisol responses may not be accurate indicators of nociceptive pain, as already discussed. The authors of the study mentioned that previous studies have suggested an onset of 20 min for procaine, and that most work evaluating the efficacy of procaine used a 10 to 30 min interval between drug administration and surgery (30). Interestingly, guidelines given to Danish farmers, and based on recommendations from the manufacturers of Procamidor^®^ Vet., recommend an interval of 5 to 10 min. This timing is in line with our results, showing the best efficacy after 5 to 10 min compared to the other intervals examined. This gap between the interval commonly used in the literature and the interval recommended in practice could be explained by potential discrepancies between pre-existing knowledge on the low diffusing ability and onset of action of procaine (26), and the formula and characteristics of specific procaine-based products available commercially. Considering the relatively low rate of diffusion of procaine (24), it cannot be excluded that an interval inferior to 5 min does not result in sufficient neuronal blocking. In addition, a study using lidocaine injected intra-testicularly showed a 90% reduction in concentration of the product in the testes between 3 and 40 min post-injection, with a very low concentration in the spermatic cord at 40 min, suggesting that a relatively long interval between injection and castration would reduce the anesthetic efficacy (41). Even with a potential lower rate of diffusion, a similar effect may be seen for procaine as of 30 min post-injection. An interval of 5 to 10 min between the administration of procaine and surgical castration therefore seems preferable as compared to 2.5 or 30 min. In practice, this interval also presents the advantage of reducing the overall duration of handling, as compared to the 20 or 30 min used the literature, limiting the time spent away from the sow and home pen, and resulting stress and heat loss.

Overall, our results showed that injection of procaine as a local anesthetic allows some mitigation of piglets' acute responses to castration, but leads to significant responses during the injection of the anesthetic. Piglets in the present study did not receive any analgesic [as is mandatory in Denmark (19)] until after data collection was over, as our aim was to assess the efficacy of the local anesthetic, and because the comparison of the different intervals would have been confounded by the use of an analgesic given at a specific time before castration. In practice, however, analgesics are administered only few minutes before castration or at the same time, and are therefore likely only efficient in mitigating post-castration pain and not the acute response to castration, as examined in the current study (42).

In the future, the use of local anesthetics prior to piglet castration in Europe may be further improved by the approval of other local anesthetics than procaine, such as lidocaine, which may have an increased efficacy at castration compared to procaine (23). Yet, in both cases, the procedure requires needle injection and extra handling, which, in our study, resulted in clear stress responses, even in piglets not exposed to any tissue damage but kept in a castration bench.

In conclusion, while our study provided further insights into the administration of a procaine-based local anesthetic prior to piglet castration, and has provided results allowing a refinement of the procedure, we cannot oversee that castration with local anesthesia remains a welfare concern. This concern has been raised previously [e.g., (11, 12, 27)], and is in line with the ‘European Declaration on alternatives to surgical castration in pigs', a document produced after consultation of various stakeholders, judging castration with anesthesia as an unsuitable alternative (16). Thus, further research into the practical application of alternatives to surgical castration or alternatives to the use of local anesthesia is still recommended.

## Data availability statement

The original contributions presented in the study are included in the article/Supplementary material, further inquiries can be directed to the corresponding author.

## Ethics statement

The animal study was reviewed and approved by the Danish Animal Experiments Inspectorate. Written informed consent was obtained from the owners for the participation of their animals in this study.

## Author contributions

The study was designed by the project group involving MH, JM, MC, and MK with advice from LF. MK developed the anesthesia injections and trained the herdsman. MC was in charge of the data collection, data processing, and data analysis with supervision from JM, LF, and MH. LF performed the sample size calculations. MC drafted the manuscript. All authors contributed to the article and approved the submitted version.

## Funding

This study was commissioned and funded by the Danish Veterinary and Food Administration (DVFA) as part of the agreement with Aarhus University on research-based policy support, as well as by Aarhus University. The funders had no role in study design, data collection and analysis, decision to publish, or preparation of the manuscript.

## Conflict of interest

The authors declare that the research was conducted in the absence of any commercial or financial relationships that could be construed as a potential conflict of interest. The reviewer ND declared a past collaboration with one of the authors MH to the handling Editor.

## Publisher's note

All claims expressed in this article are solely those of the authors and do not necessarily represent those of their affiliated organizations, or those of the publisher, the editors and the reviewers. Any product that may be evaluated in this article, or claim that may be made by its manufacturer, is not guaranteed or endorsed by the publisher.

## Supplementary material

Supplementary Video S1 Video of the intra-testicular injection procedure.

Supplementary Dataset S1 Dataset used for statistical analysis.

Supplementary Figure S1 The adapted castration bench used during injection, castration and sham handling. Picture of the bench **(1a)**, with microphone for vocalisation recording **(1b)**, camera for recording of the resistance movements **(1c)**, and soft layer matt ensuring the fitting of each piglet during the procedures **(2)**.

Supplementary Text S1 Description of the parameters used for calibration of the band limited energy automatic detection of calls in the Raven Pro software.

Supplementary Table S1 Saliva cortisol concentrations recorded for each sampling time.
