# Supplementary material for: Piglets' acute responses to local anesthetic injection and surgical castration: Effects of the injection method and interval between injection and castration
Source: Front Vet Sci. 2022 Sep 29;9:1009858. doi: 10.3389/fvets.2022.1009858 (PMC9556771; doi:10.3389/fvets.2022.1009858)
Supplement: Supplementary Video S1 — Video of the intra-testicular injection procedure. [file Data_Sheet_1.zip › Table S1.PDF]

S1 Table. **Saliva cortisol concentrations recorded for each sampling time**

		IF02	IF05	IF10	IF30	IT02	IT05	IT10	IT30	CC	SH02	SH05	SH10	SH30	P
Saliva Cortisol (pg/mL)	1	9,573 ±966 (n=50)	10,490 ±960 (n=49)	10,534 ±1,172 (n=50)	9,841 ±952 (n=50)	9,150 ±823 (n=49)	8,055 ±622 (n=48)	9,940 ±802 (n=49)	8,898 ±698 (n=48)	10,311 ±920 (n=49)	9,564 ±855 (n=50)	11,203 ±1,606 (n=25)	9,149 ±955 (n=25)	9,381 ±624 (n=49)	n.s.
	2	16,958 ±1,100 (n=50)	16,398 ±1,196 (n=49)	19,132 ±1,289 (n=50)	19,713 ±1,325 (n=49)	17,518 ±1,277 (n=50)	16,291 ±816 (n=48)	19,605 ±1,482 (n=49)	17,207 ±936 (n=49)	15,057 ±858 (n=49)	18,589 ±1,255 (n=50)	18,742 ±1,750 (n=25)	13,687 ±1,289 (n=25)	15,842 ±1,053 (n=49)	<0.01 *
	3	9,339 ±1,098 (n=50)	10,132 ±895 (n=49)	10,446 ±722 (n=50)	9,558 ±1,218 (n=49)	10,743 ±1,098 (n=50)	8,813 ±641 (n=48)	9,740 ±875 (n=49)	8,902 ±517 (n=48)	9,060 ±624 (n=49)	8,857 ±771 (n=50)	10,450 ±1,492 (n=25)	8,458 ±394 (n=25)	8,655 ±472 (n=49)	n.s.

1, sampling 1 (baseline); 2, sampling 2 (17 min after intervention); 3, sampling 3 (6 h after intervention); IF, intra-funicular injection; IT, intra-testicular injection; SH, sham handling; CC, castration without pain mitigation (control-castrated).
