# Supplementary material for: Piglets' acute responses to local anesthetic injection and surgical castration: Effects of the injection method and interval between injection and castration
Source: Front Vet Sci. 2022 Sep 29;9:1009858. doi: 10.3389/fvets.2022.1009858 (PMC9556771; doi:10.3389/fvets.2022.1009858)
Supplement: Supplementary Video S1 — Video of the intra-testicular injection procedure. [file Data_Sheet_1.zip › Text S1.PDF]

S1 Text. Description of the parameters used for calibration of the band limited energy automatic detection of calls in the Raven Pro software.

Groups of recordings were opened together as one page sound in RavenPro. Depending on the number of piglets tested each experimental day (i.e. length of the resulting page sound), either a third, half, or all recordings for one testing day were processed together.

The band limited energy detector function was applied to the entire resulting sound, using the parameters displayed in S1 Table. The parameters used were defined in accordance with the guidelines explained in the Raven Pro 1.4 User's Manual (Charif et al., 2010), and adjusted after testing on 30 random vocal files. A comparison of calls recorded with the automatic detection tool and manually selected using auditory and visual cues from the spectrogram and waveform showed a Pearson's correlation coefficient of 91% in terms of numbers of calls detected and of 77% in terms of total duration of call detected. Despite efforts to calibrate the automatic detection tool to avoid the selection of noises such as clicking of the castration bench and experimenters‘ voices, both were recurrently detected as calls. A manual check of each file was therefore implemented to review the call detected.

Type of parameter	Parameter	
**Target**	Min frequency (Hz)	1000
	Max frequency (Hz)	40000
	Min duration (s)	0.2
	Max duration (s)	3
	Min separation (s)	0.03
**Noise**	Min occupancy (%)	25
	SNR threshold (dB)	10
	Block size (s)	15
	Hop size (s)	1

**Noise Power Estimation Parameters**	Percentile (%)	25
**Exclusion Band**		None

A batch analysis function is available on Raven Pro, and should allow application of the band limited energy detector to a large number of vocal files at once, without requiring to merge certain files together. However, this function was defective at the time of the data analysis, and could therefore not be used in this study.
